# Traditional and non-traditional treatments for autism spectrum disorder with seizures: an on-line survey

**DOI:** 10.1186/1471-2431-11-37

**Published:** 2011-05-18

**Authors:** Richard E Frye, Swapna Sreenivasula, James B Adams

**Affiliations:** 1 Department of Pediatrics, University of Texas Health Science Center, Houston, USA; 2 School of Public Health, University of Texas Health Science Center, Houston, USA; 3 School of Mechanical, Aerospace, Chemical and Material Engineering, Arizona State University, Tempe, USA

## Abstract

**Background:**

Despite the high prevalence of seizure, epilepsy and abnormal electroencephalograms in individuals with autism spectrum disorder (ASD), there is little information regarding the relative effectiveness of treatments for seizures in the ASD population. In order to determine the effectiveness of traditional and non-traditional treatments for improving seizures and influencing other clinical factor relevant to ASD, we developed a comprehensive on-line seizure survey.

**Methods:**

Announcements (by email and websites) by ASD support groups asked parents of children with ASD to complete the on-line surveys. Survey responders choose one of two surveys to complete: a survey about treatments for individuals with ASD and clinical or subclinical seizures or abnormal electroencephalograms, or a control survey for individuals with ASD without clinical or subclinical seizures or abnormal electroencephalograms. Survey responders rated the perceived effect of traditional antiepileptic drug (AED), non-AED seizure treatments and non-traditional ASD treatments on seizures and other clinical factors (sleep, communication, behavior, attention and mood), and listed up to three treatment side effects.

**Results:**

Responses were obtained concerning 733 children with seizures and 290 controls. In general, AEDs were perceived to improve seizures but worsened other clinical factors for children with clinical seizure. Valproic acid, lamotrigine, levetiracetam and ethosuximide were perceived to improve seizures the most and worsen other clinical factors the least out of all AEDs in children with clinical seizures. Traditional non-AED seizure and non-traditional treatments, as a group, were perceived to improve other clinical factors and seizures but the perceived improvement in seizures was significantly less than that reported for AEDs. Certain traditional non-AED treatments, particularly the ketogenic diet, were perceived to improve both seizures and other clinical factors.

For ASD individuals with reported subclinical seizures, other clinical factors were reported to be worsened by AEDs and improved by non-AED traditional seizure and non-traditional treatments.

The rate of side effects was reportedly higher for AEDs compared to traditional non-AED treatments.

**Conclusion:**

Although this survey-based method only provides information regarding parental perceptions of effectiveness, this information may be helpful for selecting seizure treatments in individuals with ASD.

## Background

Individuals with autism spectrum disorder (ASD) have a 3 to 22-fold increase in the risk of developing epilepsy as compared to typically developing individuals and up to 25% of individuals with ASD will experience a clinical seizures at some point in their life [1,2]. However, the relationship between epilepsy and ASD is complex [3]. For example, a significant number of individuals with ASD manifest epileptiform abnormalities on electroencephalograph (EEG) despite a lack of clinical seizures, and many of these epileptiform abnormalities do not meet criteria for electrographic seizures [4].

Despite the high prevalence of seizure, epilepsy and abnormal EEGs in individuals with ASD, there is little information regarding the relative effectiveness of treatments for epilepsy, seizure or subclinical epileptiform discharges in this population. There is good reason to believe that specific antiepileptic drugs (AEDs) might be effective for individuals with ASD. For example, ASD is associated with cortical hyperexcitability, potentially due to deficits in cortical inhibitory circuits [5]. This suggests that AEDs that enhance gamma-aminobutyric acid pathways might be relatively better treatments for individuals with ASD. Additionally, the non-seizure effects of AEDs, such as mood regulation, could be particularly helpful in individuals with ASD [6]. Finally, older AEDs tend to have higher rates of adverse effects, particularly with respect to attention, mood and cognition [7,8]. Since individuals with ASD already have problems with attention, mood and cognition, prescribing an AED with such adverse effects might result in poor overall function even if seizures are controlled. Thus, the first purpose of this study was to determine whether there are specific AEDs that are more appropriate to use in children with ASD.

Many individuals with ASD use non-traditional treatments, such as special diets and nutritional supplements [9,10]. Some non-traditional treatments may help with the frequency and severity of seizures since many of these treatments target inflammation and oxidative stress [9,11,12], two pathological processes believed to be involved in the pathogenesis and propagation of seizures [13-15]. Thus, a second purpose of this study was to determine whether there are any non-traditional treatments that may be effective for treating seizures in children with ASD.

To shed light on the ability of treatments used in ASD to affect seizures and other important clinical characteristics associated with ASD, we developed an on-line seizure survey to investigate parental perceptions about the effect of treatments for their children with ASD on seizures. This survey included questions on seizure characteristics, comorbid medical conditions, the effect of traditional AED and non-AED seizure treatments and non-traditional ASD treatments on seizures, behavior and cognition, and adverse effects of treatments. The survey was designed to keep the completion time to less than 30 minutes. Although parental reports have their limitations, seizure treatment is often an individualized trial-and-error process, thus, it was the third purpose of this study to provide data that may help clinicians select treatments which are more likely to reduce seizures with minimal side-effects, and to help increase the awareness of treatment side-effects.

## Methods

### Institutional Review Board Approval

This study was conducted in accordance with the Declaration of Helsinki and the Institutional Review Board. Since the survey was anonymous and did not contain any unique indentifying information or protected health information, the study qualified for category 2 exempt status according to 45 CFR 46.101(b). Information regarding Institutional Review Board approval and contact information was provided on the first page of the survey.

### Survey Development

The first and last authors drafted an invitation letter and survey. The survey was designed using the principles outlined by Keenan [16] as implemented with the SurveyMethods, Inc. website software (http://www.SurveyMethods.com). Straightforward unambiguous non-open-ended questions were used when possible. The survey was designed to be comprehensive in the variety of possible responses so as to eliminate the need for free text entry. In order to reduce perceived bias of the survey for a specific type of treatment, the survey was designed to include a wide range to treatments commonly used in the treatment of ASD, both traditional treatments for seizures as well as non-traditional treatments.

The first page of the survey provided the responder with basic information regarding the purpose of the survey, a statement regarding the host institution and regulatory approval from the institution and an approximate completion time. The second page of the survey provided the responder with more specific information regarding the structure of the survey, the correct manner in which to respond to specific questions and contact information for the principal investigator if the responder wanted more information or had any questions. The survey did not record any identifying information of the responder nor did it ask for any protected health information identifiers. The survey was designed to be easy to answer with validation checks for each response and used skipping logic to make the survey easy and efficient to answer. Except where noted, answers were providing by multiple checkbox, rating scales, yes/no questions or specific fill in the blank questions. Very few open-ended questions or questions that required free text entry were used.

Some basic information was collected about each child with ASD including current age, gender, spectrum diagnosis and developmental profile, commonly associated medical conditions (a text box was provided for entering other medical conditions not listed), and the practitioners that manage the child's medical and developmental disorders.

Specific information regarding seizures was also queried. Responders indicated practitioners that managed or rule-out the seizure disorder and the type of test used to diagnose or rule out seizures. Responders indicated whether the individual with ASD had any of the following seizure types: generalized, partial complex, absence, typical or atypical Landau-Kleffner syndrome, subclinical epileptiform discharges, Lenox-Gastaut syndrome and/or infantile spasms. These choices were provided in a checkbox fashion so that multiple seizure types could be indicated. Next to each common seizure type was a detailed description of the seizure type to help the responder select the correct seizure type. There was also a checkbox for the responder to place other seizure types if the seizure types listed were not sufficient. Other information regarding seizures (including age of onset and age of resolution of seizures if the seizures resolved) was also collected.

Following collection of this basic descriptive information, the respondents were asked to indicate if their child had been treated with a wide range of treatments. Information for each treatment was collected in a sequential manner. For each treatment, a page would appear with a yes/no question asking the respondent whether a specific treatment had been used. Both generic and brand names, inclusive of all known brand names, were included in the question. If the respondent answered 'yes' they were directed to a page where they could rate the perceived effect of the treatment and list the adverse effects. If they answered 'no' the respondent was questioned about the next treatment. This skip logic in the software eliminated the need for the respondent to understand which questions needed to be answered and which needed to be skipped, thereby eliminating potential confusion that can occur with conditional questions in surveys. Additional pages asked the respondent if their child was treated for seizures with a treatment that was not mentioned. If the respondent answered 'yes', a page appeared which included a text box to enter the information regarding the treatment along with a page to rate the perceived effect of the treatment and any adverse effects.

For each treatment, the respondent was asked to rate the perceived effect of the treatment on seizures, sleep, receptive and expressive language, verbal and non-verbal communication, stereotyped/repetitive movements, rigidity, hyperactivity, attention and mood. A seven point scale was used that ranged from a substantial negative effect, a moderate negative effect, to a mild negative effect to no effect to a mild positive effect, a moderate positive effect, to a substantial positive effect. All ratings for this complex multi-dimensional construct of the perceived treatment effect were included on the same page to facilitate the respondent's use of the same psychometric scale. The rating scale was designed to be symmetric (i.e., same number of positive and negative ratings) in order to minimize response bias. Three sets of text boxes were provided to enter any adverse effect, the severity of the adverse effect and the frequency of the adverse effect. This allowed the respondent to enter up to three adverse effects.

### Survey Validation

The survey and participant invitation letter were developed with the advice of a wide variety of experts with experience in the treatment of children with ASD. A copy of the invitation letter and a link to the initial survey were sent to participants of the Elias Tembenis Seizures Think Tank (which took place at the AutismOne Meeting in Chicago in May of 2009) approximately one month before the think tank. The participants of this think talk represented a wide variety of practitioners who treat ASD (See See Additional file 1, Appendix A). Participants were asked to complete the survey as many times as necessary to get familiar with the survey. During the day-long think tank the first and last authors led a discussion querying the participants on their opinion regarding the specific information about the children with ASD and seizures, the treatments that should be surveyed and the specific effect of each treatment that should be asked as well as the wording of the survey. Over the weeks following the survey, changes were made to the initial survey and invitation letter as a result of the suggestions of the members of the think tank. Participants were again asked to complete the survey and provide additional comments by email. The survey was again modified and the participants were again asked to review the survey. After no further suggestions were made, the first and last authors recruited volunteer parents with children affected by ASD and seizures to review the survey and invitation letter. The survey and invitation letter were sent to these volunteers and their suggestions were integrated into the survey and letter and the modified survey and letter were again sent out for review to these volunteers. After no more significant comments were received, the final survey and invitation letter were prepared.

### Treatments Surveyed

The expert group decided on including the following treatments for the survey. Traditional seizure treatments included valproic acid, phenytoin, lamotrigine, levetiracetam, caramazapine, topiramate, oxcarbazepine, pyridoxine, clonazepam, phenobarbitol, zonisamide, gabapentin, felbamate, ethosuximide, tigabine, primidone, vigabatrin, neurofeedback, ketogenic diet, Atkin's or modified Atkin's diet, steroids, vagus nerve stimulation, surgery, intravenous immunoglobulin, transcranial magnetic stimulation/direct current stimulation. Non-traditional treatments included gluten free casein free diet, specific carbohydrate diet, hyperbaric oxygen therapy, 5-Hydroxytryptophan, gamma-aminobutyric acid, dimethylglycine, taurine, chelation therapy, co-enzyme Q10, B6, gluatathione, magnesium, B12, L-carnitine/Acetyl-L-carnitine, L-carnosine, minocycline, bacopa, actos, and spironolactone.

Although children were likely provided multiple treatments at the same time, information regarding response to specific treatment was queried individually for each treatment. This assumes that each treatment is having an influence independent of the other treatments. The authors and the expert group believe this was a necessary limitation since asking about each combination of treatments would create a questionnaire that would be prohibitively long and complex. In addition, it is likely that the number of respondents with experience with specific treatment combinations would be prohibitively small for a valid analysis. From a practical point of view, most practitioners usually start and/or stop treatments independent each other so that the clinical effect (and adverse effect) can be determined for the specific treatment.

### Questions Included in the Survey But Not Addressed In This Study

In addition to questions regarding seizures, the survey contained a small section in the beginning that asked about the effect of allergies and season on seizures and behavior. Additional questions within the survey also asked about over- or under-reactivity to external stimuli and the effect of treatments on such reactivity. These aspects of the survey are not addressed in this manuscript.

### Control Survey

In order to determine a baseline for the data collected on children with ASD and seizures, a control survey was developed to gather information regarding children with ASD without seizures (henceforth described as the control survey). This control survey contained all of the questions that the seizure survey contained except for specific questions about seizures (e.g., "What type of seizures has your child been diagnosed with?"). All treatments in the seizure survey were included in the control survey but a rating for the effect of the treatment on seizures was not included. Questions were included regarding whether the child had been evaluated for seizure, what type of practitioner evaluated the child and what test, if any, had been done to rule-out seizures.

### Recruitment

An invitation letter (See See Additional file 1, Appendix B) for the on-line survey was posted on the website and in email newsletters of the Autism Research Institute (ARI) and approximately 30 local and national ASD support groups for parents of individuals with ASD. The non-ARI support groups included Autism Speaks, several local chapters of the Autism Society of America and ARC and other local support groups for families of autism. The letter specifically asked parents of children with ASD both with and without seizures to follow one of two web links depending on whether or not their child had clinical seizures, subclinical epileptiform discharges or seizure-like activity. These web links activated different surveys located on the SurveyMethods, Inc. website (http://www.SurveyMethods.com). Identical letters were used for ARI and non-ARI websites except that the links referred to surveys that stored responses in different databases. This allowed responses to exact same seizure or control survey questions to be stored in different databases depending on whether the respondent had followed the link from the ARI or non-ARI webpage.

### Survey Response Reduction

The frequencies of specific genetic conditions were very low so all responses for specific genetic disease were included as a general genetic condition response. Treatments with less than 20 total responses were excluded from all analyses. These included surgery, transcranial magnetic stimulation/direct current stimulation, tigabine, primidone, vigabatrin, neurofeedback, minocycline, bacopa, actos, and spironolactone. Treatments with less than 20 responses for the subclinical seizure group were excluded from the subclinical seizure treatment analysis. These included ethosuximide, phenytoin, clonazepam, gabapentin, zonisamide, felbamate, phenobarbitol, vagus nerve stimulator, intravenous immunoglobulin, hyperbaric oxygen therapy, dimethylglycine, gamma-aminobutyric acid, and specific carbohydrate diet. For the subclinical seizure treatment analysis, responses for the ketogenic diet and Atkin's or modified Atkin's diet were combined because of their similarity in order to prevent elimination due to too few responses.

### Statistical Analysis

Chi-squares were used to analyze bivariate variables. To mitigate the effect of multiple comparisons, for each set of comparisons made, the Bonferroni correction was calculated to correct the alpha cutoff. The caption of each table explains the appropriate Bonferroni correction.

Ratings were converted into an ordinal scale ranging from 1 to 7 for analysis: substantial negative effect (1), a moderate negative effect (2), a mild negative effect (3), no effect (4), a mild positive effect (5), a moderate positive effect (6), and a substantial positive effect (7). Although the response scale was ordinal, the response distribution was found to be normally distributed allowing the use of parametric analyses for treatment ratings.

Comparing every treatment to every other treatment would result in a very large number of comparisons (>500), thereby considerably increasing the probability of a type I error. To reduce the number of comparisons we investigated whether multiple treatments could be clustered together into treatments that showed the same pattern of ratings for seizures and other clinical factors (i.e., sleep, communication, behavior, attention, mood) and then compared the ratings from each treatment cluster to other treatment clusters.

For the cluster analysis, seizure types were divided into two broad categories: clinical (generalized, partial complex, absence) and subclinical (typical and atypical Landau-Kleffner syndrome, subclinical epileptiform discharges). Lenox-Gastaut syndrome and infantile spasms were not considered in this manuscript. For the cluster analysis, two summary scales were calculated to reduce the number of rating scales: receptive and expressive language and verbal and non-verbal communication ratings were averaged to create a rating called communication and stereotyped/repetitive movements, rigidity and hyperactivity ratings were averaged to create a rating called behavior. For subclinical seizures we did not include the seizure ratings in the cluster analysis as the primary manifestations of subclinical seizures are other clinical factors and seizures are not reliably detected in individuals with subclinical seizures.

Cluster analysis was conducted using Ward's technique [17]. The Ward's technique defines the distance between treatments in terms of the between cluster variability to the within cluster variability. The Ward's technique is a hierarchical analysis that starts with *n *clusters, one for each treatment, and then at each step groups the most similar treatments into clusters. This procedure continues until there is one cluster containing all respondents. By examining the dendogram and several statistics (pseudo F and t), a judgment is made about the number of clusters [18].

The ratings of treatments within each cluster were compared to the rating from other clusters using a mixed-model analysis-of-variance (ANOVA) with two fixed-effects: cluster and seizure type (generalized, partial complex, absence for clinical seizures and Landau-Kleffner syndrome, atypical Landau-Kleffner syndrome, subclinical epileptiform discharges for subclinical seizures), and the interaction between these factors. The ANOVA was calculated using the 'glimmix' procedure of SAS 9.1 (SAS Institute Inc., Cary, NC) with respondent and seizure type as a random variable. Seizure type and the interaction were not significant in any analysis. Statistical values for the analysis of variables are presented in supplementary tables (See Additional file 2) along with the calculation for the Bonferroni correction. The statistical values for the seizure type and the interaction effects were not included in the tables since they were not significant. For selected clusters, individual treatments were compared using a similar ANOVA.

Planned contrasts were used to compare ratings. Planned contrasts were calculated using the 'estimate' command in SAS for the 'glimmix' procedure. The procedure uses both the fixed-effects and random-effects matrices to construct a matrix with an approximate t distribution. The unadjusted t-values and p-values are presented and the Bonferroni correction was used to calculate the appropriate alpha levels for each set of comparisons. Statistical values for the cluster or treatment effects of the analysis are presented in supplementary tables (See Additional file 2), along with the results of the contrasts.

## Results

### Characteristics of seizure and control groups

Overall, 1023 responders completed the surveys, with 733 responses concerning children with ASD and clinical seizures, subclinical epileptiform discharges or seizure-like activity and 290 control responses. Seven invalid responses were deleted.

In both the seizures and control surveys, 77% of the children were male. This proportion was not significantly different across control and seizure survey groups. Children in the control survey were significantly younger [9y 5m (SD 5y 11m)] than the children in the seizure survey [13y 4m (SD 6y 8m); t = 9.0, p < 0.0001]; this may relate to some individuals only developing seizures later in life. Table 1 outlines the practitioners who regularly managed children reported in the control and seizures surveys. Overall, the majority of children were managed, at least in part, by a pediatrician. The second most prevalent practitioner was a child neurologist, although this proportion was significantly higher for the children with seizures as compared to the controls. One-third of children were managed, at least in part, by a doctor affiliated with Defeat Autism Now! (an educational program of the ARI) and one-fifth of children were managed, at least in part, by a psychiatrist. Children were managed by family and general practitioners, at least in part, 15% and 14% of the time, respectively. Children were managed, at least in part, by holistic and integrative medicine practitioners 10% and 5% of the time, respectively. An adult neurologist managed, at least in part, 11% of children but this proportion was significantly higher for the seizures group as compared to the control group. Indeed, almost none of the children in the control group were managed by an adult neurologist. These proportions were not different for those who responded to the ARI invitation as compared to the support group invitations.

**Table 1:** Practitioners who regularly manages the child with ASD.

Practitioner	Overall	Controls	Seizures
Pediatrician	66%	59%	69% ^ns^

Child Neurologist	49%	23%	60%^†^

Doctor affiliated with Defeat Autism Now!	33%	29%	34%^ns^

Psychiatrist	20%	20%	20% ^ns^

Family practitioner	15%	14%	15% ^ns^

General practitioner	16%	10%	18% ^ns^

Adult Neurologist	11%	0%	15%^†^

Holistic Medicine	10%	9%	10% ^ns^

Integrative Medicine	5%	5%	5%^ns^

‡p < = 0.001; †p < = 0.0001

For each practitioner listed, statistical comparisons were made between the individuals reported to have seizures and the individuals reported not to have seizures (control group). Superscript after the seizure percentage indicates whether the proportions are different between the two groups. There are 9 practitioners resulting in 9 comparisons. The Bonferroni correction results in an alpha of 0.05/9 = 0.0056, so we have set the alpha to p < = 0.001 to be conservative.

Fifty-three respondents reported that the child had both clinical and subclinical seizures. These responses were included in both the clinical and subclinical seizure groups. This resulted in 548 responses about children with clinical seizures and 144 responses about children with subclinical seizures. Males made up 76% and 78% of the clinical seizures and subclinical seizure groups, respectively, with no significant difference in these proportions across groups. The average age of the child at the time the survey was 13y 5m (SD 7y 1m) and 12y 1m (SD 6y 6m) for children with clinical and subclinical seizures, respectively. This age difference was not statistically significant. Seizures resolved in 15.8% and 16.0% of the children reported to have clinical and subclinical seizures, respectively. In children with resolved seizures, clinical and subclinical seizures were reported to start at 5y 7m (SD 5y 11m) and 5y 11m (SD 6y 5m), respectively, and resolve at 9y 7m (SD 6y 7m) and 10y 11m (SD 6y 6m), respectively. These ages were not significantly different between groups. For children with seizures that did not resolve, seizures were reported to start at a slightly younger age (t = 3.70, p < 0.001) for those reported to have subclinical seizures [5y 11m (SD 4y 4m)] as compared those reported to have clinical seizures [6y 2m (SD 5y 7m)]. The length of time a child was affected by seizures was 4y 0m (4y 3m) and 5y 0m (4y 0m) for those whose seizures had resolved for clinical and subclinical seizures, respectively and the length of time a child was affected by seizures was 7y 2m (6y 7m) and 7y 3m (6y 2m) for those whose seizures had not resolved for clinical and subclinical seizures, respectively. The length of time was not significantly different between the clinical and subclinical seizures groups for children whose seizures were reported resolved or reported not to resolve.

Table 2 outlines the type of practitioner who diagnosed and managed the seizures in children reported in the survey organized by practitioner prevalence as calculated by the weighted average of the three groups. For the control survey this question pertained to the practitioner who performed an evaluation to rule-out clinical or subclinical seizures. Since some controls might not have been evaluated for seizures, an additional option was included in the control survey to indicate that no evaluation had been performed. A child neurologist most often diagnosed and managed seizures, on average, with the percentage of children with clinical or subclinical seizures being diagnosed and managed by a child neurologist significantly more often than controls. In fact, the great majority of children with clinical or subclinical seizures were diagnosed and managed by a child neurologist as compared to other practitioners. Pediatricians were the 2^nd ^most likely practitioner to diagnose and manage seizures but this was primarily due to the high rate of control children that were evaluated by pediatricians. In fact, a pediatrician evaluated the great majority of control children. Doctors affiliated with Defeat Autism Now! were the 3^rd ^most likely practitioners reported, on average, to diagnose and manage seizures. Doctors affiliated with Defeat Autism Now! were less likely to diagnosed and manage seizures in children with clinical seizures as compared to children with subclinical seizures or control children. Adult neurologists were the 4^th ^most likely practitioners, on average, to diagnose and manage seizures but this was primarily due to the significantly higher rate of children with clinical or subclinical seizures being managed by an adult neurologist. Psychiatrists and family medicine, holistic medicine and integrative medicine practitioners were the 5^th^, 6^th^, 7^th ^and 8^th ^most likely practitioners to diagnose and manage seizures. These practitioners were more likely to evaluate control children than diagnose and manage children with clinical or subclinical seizures. General practitioners managed very few children reported by the respondents of this survey.

**Table 2:** Practitioners who evaluated and managed ASD individual for seizures.

Practitioner	Overall	Controls	Clinical Seizures	Subclinical Seizures
Child Neurologist	58.4%	30%	71%^†^	76% ^†,ns^

Adult Neurologist	12.7%	2%	19%^†^	12%^†,ns^

Doctor affiliated with Defeat Autism Now!	10.9%	2%	13%^†^	21%^†,ns^

Psychiatrist	9.3%	2%	5%^†^	4%^†,ns^

Pediatrician	7.2%	4%	9%^†^	9%^†,ns^

Family practitioner	3.7%	1%	3%^†^	3%^†,ns^

Holistic Medicine	3.0%	0%	4%^†^	5% ^†,ns^

General practitioner	1%	1%	1%^†^	1%^‡,ns^

Integrative Medicine	0.7%	0%	1%^ns^	1% ^ns,ns^

No Evaluation		60%		

‡p < = 0.001; †p < = 0.0001

For each practitioner listed, statistical comparisons were made between the clinical seizure group and the control group (superscript after the clinical seizure percentage), the subclinical seizure group and the control group (1st superscript after the subclinical seizure percentage) and between the clinical and subclinical seizure group (2nd superscript after the subclinical seizure percentage). There are 9 practitioners and 3 comparisons for each practitioner resulting in 27 comparisons. The Bonferroni correction results in an alpha of 0.05/27 = 0.0019, so we have set the alpha to p < = 0.001 to be conservative. Note that the seizures groups were not given the option of answering no evaluation so percentages are not reported.

Table 3 outlines the tests used to diagnose or rule-out seizures in children with ASD. The diagnostic tests are organized by the overall percentage of children who received such tests as calculated by the weighted average of the three groups. A routine EEG was the most frequently used diagnostic test with significantly more children with clinical and/or subclinical seizures having had a routine EEG as compared to controls. The overnight EEG was the second most widely used diagnostic test with significantly more children with clinical and/or subclinical seizures having had an overnight EEG as compared to controls. In addition, significantly more children with subclinical seizures received an overnight EEG as compared to children with clinical seizures. A minority of children diagnosed with clinical and/or subclinical seizures were diagnosed without a diagnostic test, while just over half of the controls did not receive a diagnostic test - a percentage similar to the proportion of controls that were not evaluated for seizures. An ambulatory EEG was the third most often used test for all groups, although it was used significantly less in the control group. Magnetoencephalography, positron emission tomography and single photon emission computed tomography were used in a small percentage of the control group and in a minority of the clinical and subclinical seizure groups.

**Table 3:** Tests used to diagnose or rule-out seizures by seizure group.

Diagnostic Test	Overall	Controls	Clinical Seizures	Subclinical Seizures
Routine electroencephalogram	61.8%	28%	78%^†^	68%^†,ns^

Overnight electroencephalogram	34.6%	10%	41%^†^	60% ^†,†^

No Test	27.5%	56%	17%^†^	10%^†,ns^

Ambulatory electroencephalogram	17.6%	3%	21%^†^	34% ^†,‡^

Magnetoencephalography	3.4%	1%	4%^ns^	6% ^‡,ns^

Positron emission tomography	5.1%	1%	6%^‡^	10% ^†,ns^

Single photon emission computed tomography	5.5%	1%	6%^‡^	13% ^†,ns^

‡p < = 0.001; †p < = 0.0001

For each diagnostic test listed, statistical comparisons were made between the clinical seizure group and the control group (superscript after the clinical seizure percentage), the subclinical seizure group and the control group (1^st ^superscript after the subclinical seizure percentage) and between the clinical and subclinical seizure group (2^nd ^superscript after the subclinical seizure percentage). Since there are 7 diagnostic tests and 3 comparisons for each diagnostic test resulting in 21 comparisons. The Bonferroni correction results in an alpha of 0.05/21 = 0.0023, so we have set the alpha to p < = 0.001 to be conservative.

Table 4 outlines the reported spectrum diagnosis for the three seizure groups. The majority of children were reported to be diagnosed with Autism Disorder with this diagnosis reported in a higher percentage of children diagnosed with seizure or subclinical seizures as compared to controls. The second most common developmental diagnosis was pervasive developmental disorder-not otherwise specified (PDD-NOS) with the proportion of children diagnosed with PDD-NOS similar across all groups. Fewest children were diagnosed with Asperger syndrome for all groups with a significantly smaller proportion of children diagnosed with this spectrum disorder in the two seizure groups as compared to the control group. Both seizure groups were more likely to be reported to have developmental regression as compared to the control group but other developmental profiles were similar across all groups. Table 5 outlines the reported medical characteristics by seizure group. The clinical seizure group had proportionally more individuals with mental retardation as compared to the control group but all other medical disorders were similar across both seizure and control groups.

**Table 4:** Developmental Characteristics by Seizure Group.

Developmental Diagnosis	Control	Clinical Seizures	Subclinical Seizures
Autism Disorder	61%	73% ^‡^	78% ^‡,ns^

PDD-NOS	22%	19% ^ns^	16% ^ns,ns^

Asperger Syndrome	17%	8% ^†^	6% ^‡,ns^

**Developmental Profile**	**Control**	**Clinical Seizures**	**Subclinical Seizures**

Regression	18%	28% ^‡^	38% ^†,ns^

Plateau	7%	4% ^ns^	8% ^ns,ns^

Symptoms from infancy	34%	39% ^ns^	31% ^ns,ns^

No early symptoms	34%	29% ^ns^	23% ^ns,ns^

‡p < = 0.001; †p < = 0.0001

For each characteristic listed, statistical comparisons were made between the clinical seizure group and the control group (superscript after the clinical seizure percentage), the subclinical seizure group and the control group (1^st ^superscript after the subclinical seizure percentage) and between the clinical and subclinical seizure group (2^nd ^superscript after the subclinical seizure percentage). Since there are 3 developmental diagnosis categories and 3 comparisons for each diagnosis category resulting in 9 comparisons. The Bonferroni correction for developmental diagnosis portion of the table results in an alpha of 0.05/9 = 0.0056, so we have set the alpha to p < = 0.001 to be conservative. Since there are 4 developmental profile categories and 3 comparisons for each developmental profile category resulting in 12 comparisons. The Bonferroni correction for the developmental profile portion of the table results in an alpha of 0.05/12 = 0.0042, so we have set the alpha to p < = 0.001 to be conservative.

**Table 5:** Medical Characteristics by Seizure Group.

Medical Diagnosis		Overall	Control	Clinical Seizures	Subclinical Seizures
Prematurity		15%	13%	15% ^ns^	16% ^ns,ns^

Cerebral Palsy		4%	1%	5% ^ns^	3% ^ns,ns^

Sensory Integration Disorder		51%	53%	48% ^ns^	58% ^ns,ns^

ADHD		31%	31%	30% ^ns^	31% ^ns,ns^

Hypotonia		24%	20%	26% ^ns^	27% ^ns,ns^

Mental Retardation		20%	7%	28% ^†^	18% ^ns,ns^

Mitochondrial disorder		10%	6%	11% ^ns^	17% ^ns,ns^

Genetic Disorder		6%	4%	7% ^ns^	7% ^ns,ns^

Renal disease		2%	2%	2% ^ns^	1% ^ns,ns^

Cardiovascular disease		2%	2%	2% ^ns^	2% ^ns,ns^

Hematological disease		1%	1%	1% ^ns^	1% ^ns,ns^

	**Growth**				

Failure-to-Thrive		14%	11%	14% ^ns^	15% ^ns,ns^

Macrocephaly		11%	9%	11% ^ns^	13% ^ns,ns^

Accelerated Growth		10%	11%	9% ^ns^	9% ^ns,ns^

Microcephaly		4%	4%	4% ^ns^	3% ^ns,ns^

	**Gastrointestinal Disorders**				

Constipation		41%	39%	42% ^ns^	40% ^ns,ns^

GERD		22%	22%	22% ^ns^	25% ^ns,ns^

Inflammation		17%	12%	18% ^ns^	24% ^ns,ns^

Dysbiosis		16%	12%	16% ^ns^	22% ^ns,ns^

LNH		4%	2%	4% ^ns^	7% ^ns,ns^

	**Sleep Disorders**				

Disrupted Sleep		44%	34%	47% ^ns^	53% ^ns,ns^

Insomnia		22%	27%	34% ^ns^	33% ^ns,ns^

Apnea		9%	8%	9% ^ns^	9% ^ns,ns^

PLMS		6%	5%	6% ^ns^	9% ^ns,ns^

†p < = 0.0001

For each characteristic listed, statistical comparisons were made between the clinical seizure group and the control group (superscript after the clinical seizure percentage), the subclinical seizure group and the control group (1^st ^superscript after the subclinical seizure percentage) and between the clinical and subclinical seizure group (2^nd ^superscript after the subclinical seizure percentage). There are 24 medical diagnoses and 3 comparisons for each medical diagnosis resulting in 72 comparisons. The Bonferroni correction results in an alpha of 0.05/72 = 0.0007, so we have set the alpha to p < = 0.0001 to be conservative.

### Prevalence of traditional treatments in seizure and control groups

Table 6 presents the reported usage prevalence of traditional treatments for seizures organized by AED and non-AED treatments and sorted by the overall prevalence within each treatment category. Overall, AED treatments were reportedly used more often in both the clinical and subclinical seizure groups as compared to the control group, except for ethosuximide in which the usage prevalence was not significantly different for the control and clinical seizure groups. Valproic acid was the most commonly used AED for all groups and was reported to be used in almost half of the children diagnosed with clinical and subclinical seizures. Lamotrigine was the second most commonly used AED overall and was used in about one-third of patients with clinical or subclinical seizures. AED usage was reportedly similar in the clinical and subclinical seizure groups in most cases, although ethosuximide was reportedly used more often in the subclinical seizure group as compared to the clinical seizure group. Several AEDs, including lamotrigine, levetiracetam, carbamazepine, topiramate and oxcarbazepine were reportedly used in one-fourth or more of the children with clinical and/or subclinical seizures.

**Table 6:** Traditional treatment usage by seizure group.

Treatment	Overall	Controls	Clinical Seizures	Subclinical Seizures
**Anti-epileptic drug treatment**				
Valproic acid	31%	6%	39%^†^	48% ^†,ns^

Lamotrigine	22%	3%	27% ^†^	39%^†, ns^

Levetiracetam	18%	1%	23% ^†^	31% ^†,ns^

Carbamazepine	18%	1%	26% ^†^	23% ^†,ns^

Topiramate	17%	1%	25% ^†^	22% ^†,ns^

Oxcarbazepine	16%	2%	22% ^†^	24% ^†,ns^

Clonazepam	12%	3%	16% ^†^	13% ^†,ns^

Phenytoin	10%	1%	13% ^†^	13% ^†,ns^

Phenobarbital	10%	2%	14% ^†^	11% ^†,ns^

Gabapentin	7%	1%	8% ^†^	12% ^†,ns^

Zonisamide	7%	0%	10% ^†^	12% ^†,ns^

Ethosuximide	4%	0%	4% ^ns^	10% ^†, ns^

Felbamate	4%	0%	5% ^†^	7% ^†,ns^

**Non-antiepileptic drug treatments**				

Vitamin B6	19%	27%	15% ^†^	20% ^ns,ns^

Steroids	11%	21%	5% ^†^	14% ^ns, ns^

Ketogenic diet	6%	0%	7% ^†^	13% ^†,ns^

Intravenous Immunoglobulin	3%	1%	3% ^ns^	7% ^ns,ns^

Vagal Nerve Stimulator	3%	0%	4% ^ns^	6% ^ns,ns^

†p < = 0.0001

For each treatment listed, statistical comparisons were made between the clinical seizure group and the control group (superscript after the clinical seizure percentage), the subclinical seizure group and the control group (1^st ^superscript after the subclinical seizure percentage) and between the clinical and subclinical seizure group (2^nd ^superscript after the subclinical seizure percentage). There are 18 treatments and 3 comparisons for each treatments resulting in 54 comparisons. The Bonferroni correction results in an alpha of 0.05/54 = 0.0009, so we have set the alpha to p < = 0.0001 to be conservative.

Vitamin B6 and steroids were the most frequently used traditional non-AED seizure treatments. More children in the control group were treated with steroids and vitamin B6 as compared to the clinical seizure group. The ketogenic diet was the third most commonly used non-AED traditional seizure treatment. The ketogenic diet was reportedly used more often by the seizure groups than the control group.

### Prevalence of non-traditional treatments in seizure and control groups

Table 7 presents the reported usage prevalence of non-traditional treatments for seizures sorted by the overall prevalence within each treatment category. Significantly fewer children in the clinical seizure group used vitamin B12 and/or the gluten-free casein-free diet treatment as compared to the control group. Both seizure groups and the control group were reported to use all other non-traditional supplement, drug and diet treatments with a similar prevalence. Many treatments such as vitamin B12, L-carnitine/acetyl-L-carnitine and magnesium were reportedly used for approximately one-fifth of the children reported on in this survey and approximately one-third were treated, at least at some point, with the gluten-free casein-free diet.

**Table 7:** Non-traditional treatment usage by seizure group.

Treatment	Overall	Controls	Clinical Seizures	Subclinical Seizures
**Supplement treatments**				

Vitamin B12	24%	32%	20%^‡^	26% ^ns,ns^

L-Carnitine/Acetyl-L-Carnitine	21%	22%	18% ^ns^	29% ^ns,ns^

Magnesium	20%	26%	17% ^ns^	22% ^ns,ns^

Coenzyme Q10	14%	16%	12% ^ns^	15% ^ns,ns^

Glutathione	14%	14%	12% ^ns^	18% ^ns,ns^

Dimethylglycine	11%	14%	9% ^ns^	13% ^ns,ns^

Taurine	11%	10%	10% ^ns^	14% ^ns,ns^

GABA	9%	12%	8% ^ns^	9% ^ns,ns^

5-Hydroxytryptophan	8%	11%	6%^ns^	9% ^ns,ns^

L-Carnosine	6%	5%	5% ^ns^	10%^ns,ns^

**Drug treatments**				

Chelation Therapy	12%	15%	10%^ns^	16% ^ns,ns^

Hyperbaric Oxygen Therapy	7%	6%	7% ^ns^	10% ^ns,ns^

**Dietary treatments**				

Gluten Free Casein Free Diet	31%	41%	25% ^†^	31%^ns,ns^

Specific Carbohydrate Diet	6%	4%	6% ^ns^	8% ^ns,ns^

Atkins or Modified Atkins Diet	2%	1%	3% ^ns^	2% ^ns,ns^

‡p < = 0.001; †p < = 0.0001

For each treatment listed, statistical comparisons were made between the clinical seizure group and the control group (superscript after the clinical seizure percentage), the subclinical seizure group and the control group (1^st ^superscript after the subclinical seizure percentage) and between the clinical and subclinical seizure group (2^nd ^superscript after the subclinical seizure percentage). There are 15 treatments and 3 comparisons for each treatments resulting in 45 comparisons. The Bonferroni correction results in an alpha of 0.05/45 = 0.0011, so we have set the alpha to p < = 0.001 to be conservative.

### Differences in responders to the ARI and non-ARI support group invitations

Very few differences were found between the responders to the ARI and non-ARI invitations. There were no differences in age, gender, seizure type, practitioners, diagnostic tests, spectrum diagnoses or medical characteristics between responders to the ARI and non-ARI invitations. There were a few differences in the percentage of children who used certain treatments between responders to the ARI and non-ARI invitations. Responders to the ARI invitation reported using valproic acid (ARI 53% v Non-ARI 40%, χ2 = 16.18, p < 0.0001) and magnesium (ARI 25% v Non-ARI 13%, χ2 = 20.11, p < 0.0001) more often than responders to non-ARI support group invitations.

### Treatments for Clinical Seizures

**Figure 1:**
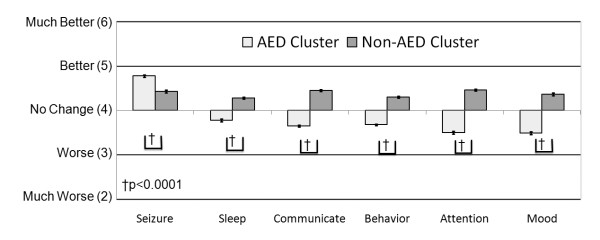
**Treatments for clinical seizures cluster into anti-epileptic drug and non-anti-epileptic drug treatments**. In general, anti-epileptic drug treatments were perceived as improving seizures significantly more than non-anti-epileptic drug treatments but significantly worsening other clinical factors.

For individuals that were reported to have clinical seizure, the average rating of the perceived effect of each treatment on seizures, sleep, communication, behavior, attention and mood was obtained. These averages were entered into a cluster analysis (discussed above in methods) to determine if certain treatments demonstrated similar effects on seizures, sleep, communication, behavior, attention and mood. The cluster analysis provided a strong separation of the treatments into two clusters: AED and non-AED treatment clusters (See Cluster 1 and Cluster 2 in Table 8). The overall average ratings for seizures, sleep, communication, behavior, attention and mood for these two clusters were obtained by averaging ratings (derived from the original ratings) across all treatments within each cluster (Figure 1). We then determined whether there was a statistical difference in the ratings between these groups by analyzing the ratings with an ANOVA that included cluster and seizure type (generalized seizures, partial seizures, absence seizures) as the independent effects as well as the interaction between these two effects. Seizure type and the interaction between seizure type and cluster were not significant. The effect of cluster was significant for all ratings, including the more specific ratings for communication and behavior (See Additional file 2, Table S1). Overall, both AED and non-AED treatments were perceived, on average, as making seizures better but treatments within the AED cluster were perceived as improving seizures significantly more than treatments in the non-AED cluster (Figure 1). On average, treatments within the AED cluster were perceived as worsening clinical factors other than seizures (i.e., sleep, communication, behavior, attention and mood) and treatments with the non-AED cluster were perceived as improving clinical factors other than seizures (Figure 1). Treatments in the non-AED cluster were perceived as improving other clinical factors significantly better than treatments in the AED cluster.

**Table 8:** Grouping of treatments for the clinical seizures group.

Antiepileptic Drugs (Cluster 1)		
**AED Subcluster 1**	**AED Subcluster 2**	**AED Subcluster 3**

Valproic acid Lamotrigine Levetiracetam Ethosuximide	Phenytoin Clonazepam Carbamazepine Oxcarbazepine Topiramate Gabapentin Zonisamide Felbamate	Phenobarbitol

**Non-Antiepileptic Drugs (Cluster 2)**		

**Non-AED Subcluster 1**	**Non-AED Subcluster 2**	**Non-AED Subcluster 3**

Ketogenic Diet Atkins Diet Gluten-free Casein-free Diet Hyperbaric Oxygen Therapy	Vitamin B6 Intravenous Immunoglobulin L-Carnitine/Acetyl-L-Carnitine CoQ10 Vitamin B12 Dimethylglycine Taurine GABA Magnesium 5HTP L-Carnosine Chelation Glutathione Specific carbohydrate diet	Steroids Vagus Nerve Stimulator

Two levels of cluster analyses were performed. The first cluster analysis (Tier 1) divided treatments into anti-epileptic medication and non-antiepileptic medications. Cluster analysis was applied to the two Tier 1 clusters separated. Three Tier 2 subclusters were found for both initial clusters.

The first (Tier 1) cluster analysis provided evidence for a significant separation between AED and non-AED treatments, but did not provide information regarding differences within each treatment cluster. In order to determine if there were differences among treatments within each cluster, a second set of cluster analyses (Tier 2) were performed on the AED and non-AED clusters separately.

#### AED Treatments

**Figure 2:**
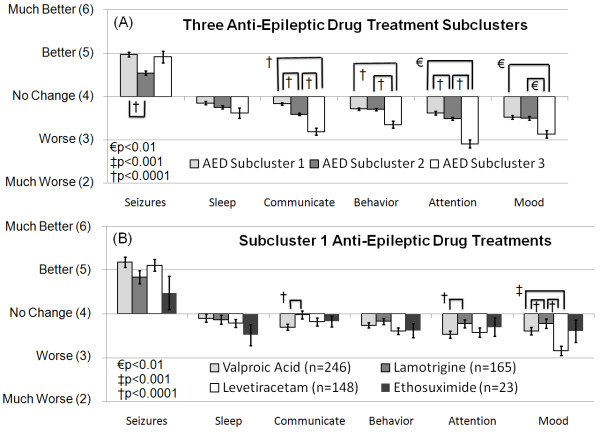
**Rating for subclusters of anti-epileptic drug treatments for clinical seizures**. (A) Anti-epileptic drug treatment subcluster 1 was perceived as improving seizures better than subcluster 2 and worsened several other clinical factors less than subclusters 2 and 3. (B) The perceived effect on seizures was not different across the four anti-epileptic drug treatments in subcluster 1. Out of the four treatments in subcluster 1, lamotrigine appeared to worse several other clinical factors, such as communication, attention and mood less than other treatments.

Cluster analysis of the AED treatments cluster resulted in three subclusters (See AED subcluster 1, 2 and 3 in Table 8). The overall average ratings for seizures, sleep, communication, behavior, attention and mood for these three subclusters were obtained by averaging ratings (derived from the original ratings) across all treatments within each subcluster (Figure 2A). It was then determined whether there was a statistical difference in the ratings between these clusters by analyzing the ratings with an ANOVA similar to the one described above. Seizure type and the interaction between seizure type and cluster were not significant. The effect of cluster was significant for all ratings, expect sleep and mood, and for the more specific ratings within communication and behavior, expect for rigidity (See Additional file 2, Table S2).

Treatments in AED subcluster 1 were perceived as improving seizures significantly more than the treatments in AED subcluster 2 but the perceived effect of the treatment in AED subcluster 3 on seizures was not significantly different than AED subclusters 1 or 2. Treatments in subcluster 1 were perceived to worsen communication and attention significantly less than treatments in AED subclusters 2 and 3 and treatments in AED subcluster 1 were perceived to worsen behavior and mood significantly less than AED subcluster 3 (Figure 1A; See Additional file 2, Table S2).

Since the treatments in AED subcluster 1 were perceived to be more beneficial overall than treatments in the other subclusters, the four treatments in AED subcluster 1 (valproic acid, lamotrigine, levetiracetam, and ethosuximide) were examined in greater detail. The overall average ratings for seizures, sleep, communication, behavior, attention and mood for these four treatments were obtained by averaging ratings from the original responses for each treatment (Figure 2B). Statistical differences in the ratings between these treatments were determined using an ANOVA similar to the one described above. Seizure type and the interaction between seizure type and treatment were not significant. The effect of AED treatment was significant for communication, attention and mood and one specific scale of behavior, rigidity (See Additional file 2, Table S3).

Overall the four AED treatments (valproic acid, lamotrigine, levetiracetam, ethosuximide) were not perceived to be different in their ability to improve seizures (Figure 2B; See Additional file 2, Table S3). Lamotrigine was perceived to worsen communication, rigidity and attention less than valproic acid and worsen rigidity and mood less than levetiracetam. Valproic acid was also found to worsen mood less than levetiracetam. Ethosuximide did not demonstrate pair-wise differences, likely due to the large variability in ratings for this treatment.

#### Non-AED Treatments

**Figure 3:**
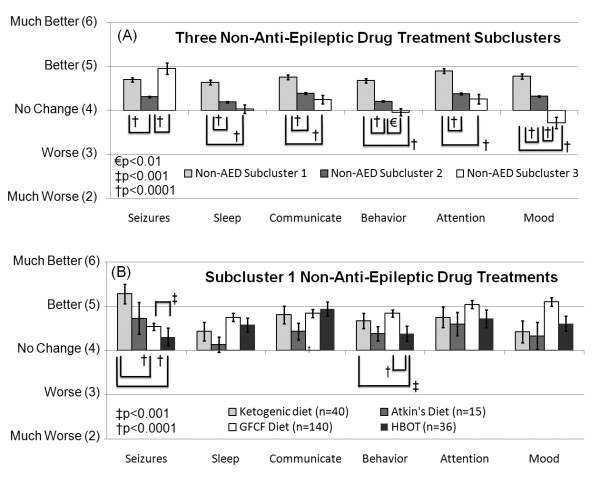
**Rating for subclusters of non-anti-epileptic drug treatments for clinical seizures**. (A) Non-anti-epileptic drug treatment subcluster 1 was perceived as improving seizures better than subcluster 2 and improving several other clinical factors more than subclusters 2 and 3. (B) The ketogenic diet was perceived to improve seizures more than the gluten-free-casein-free diet and hyperbaric oxygen treatment. Other clinical factors were not perceived to differ significantly between these four treatments except for behavior, which was perceived to be significantly worse with hyperbaric oxygen treatment as compared to the ketogenic diet and the gluten-free-casein-free diet.

Cluster analysis of the non-AED treatments resulted in three subclusters (See non-AED subcluster 1, 2 and 3 in Table 8). The overall average ratings for seizures, sleep, communication, behavior, attention and mood for these three subclusters were obtained by averaging ratings (derived from the original ratings) across all treatments within each subcluster (Figure 3A). The statistical difference in the ratings between these subclusters were analyzed using an ANOVA similar to the one described above. Seizure type and the interaction between seizure type and subcluster were not significant. The effect of subcluster was significant for all overall ratings and for the more specific ratings within communication and behavior (See Additional file 2, Table S4).

Treatments in non-AED subclusters 1 and 3 were perceived to significantly improve seizures more than treatments in non-AED subcluster 2. Treatments in non-AED subcluster 1 were perceived to improve sleep, communication, behavior, attention and mood better than treatments in non-AED subclusters 2 and 3 (Figure 3A; See Additional file 2, Table S4).

Since treatments in non-AED subcluster 1 were perceived to be more beneficial overall than treatments in the other subclusters, the four treatments in non-AED subcluster 1 (ketogenic diet, Atkin's or modified Atkin's diet, gluten-free casein-free diet, hyperbaric oxygen therapy) were examined in detail. The overall average ratings for seizures, sleep, communication, behavior, attention and mood for these four treatments were obtained by averaging ratings from the original responses for each treatment (Figure 3B). Statistical differences in the ratings between these treatments were determined using an ANOVA similar to the one described above. Seizure type and the interaction between seizure type and treatment were not significant. The effect of treatment was significant for seizures and behavior, including the subscales of behavior (See Additional file 2, Table S5).

The ketogenic diet was perceived to significantly improve seizures more than the gluten-free casein-free diet and hyperbaric oxygen therapy, and gluten-free casein-free diet was perceived to improve seizures significantly more than hyperbaric oxygen therapy (Figure 3B; See Additional file 2, Table S5). Both the gluten-free casein-free diet and the ketogenic diet were found to improve behavior significantly more than hyperbaric oxygen therapy.

### Treatments for Subclinical Seizures

**Figure 4:**
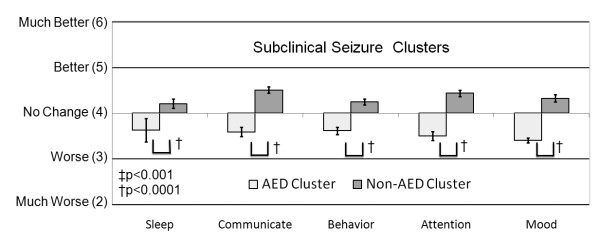
**Treatments for subclinical seizures cluster into anti-epileptic drug and non-anti-epileptic drug treatments**. In general, anti-epileptic drug treatments were perceived as worsening clinical factors while non-anti-epileptic drug treatments appeared to improve clinical factors.

For individuals reported to have subclinical seizure, the average rating of the perceived effect of each treatment on seizures, sleep, communication, behavior, attention and mood was calculated and entered into a cluster analysis to determine whether certain treatments demonstrated similar effects on sleep, communication, behavior, attention and mood. The cluster analysis provided a strong separation of the treatments into two clusters: AED and non-AED treatment clusters (See Cluster 1 and Cluster 2 in Table 9). The overall average ratings for sleep, communication, behavior, attention and mood for these two clusters were obtained by averaging ratings (derived from the original ratings) across all treatments within each cluster (Figure 4). Statistical differences in the ratings between these clusters were determined by analyzing the ratings with an ANOVA which included cluster and subclinical seizure type (typical Landau-Kleffner syndrome, atypical Landau-Kleffner syndrome, subclinical epileptiform discharges) as the independent effects as well as the interaction between these two effects. Subclinical seizure type and the interaction between seizure type and cluster were not significant. The effect of cluster was significant for all ratings, including the more specific ratings for communication and behavior (See Additional file 2, Table S6).

**Table 9:** Grouping of treatments for subclinical seizures using cluster analysis.

Treatments for Subclinical Seizures	
**Cluster 1**	**Cluster 2**

Valproic acid Levetiracetam Lamotrigine Carbamazepine Oxcarbazepine Topiramate	Vitamin B6 Steroids L-Carnitine/Acetyl-L-Carnitine CoQ10 Vitamin B12 Taurine Magnesium Chelation Glutathione Gluten-free Casein-free Diet Ketogenic Diet/Atkin's Diet

A cluster analysis divided treatments into anti-epileptic drug treatments and non-antiepileptic drug treatments. Cluster analysis was applied to clusters 1 and 2 separately but no subclusters were evident.

Sleep, communication, behavior, attention and mood were perceived to be worsened by AED treatments and improved by non-AED treatments (Figure 4; See Additional file 2, Table S6). The cluster analysis was applied to these two clusters separately but the cluster analysis demonstrated very low pseudo-F and pseudo-t values, indicating that there was no strong subclustering.

### Adverse Effects

Although the survey was designed to examine the most salient effects of treatments related to ASD, all treatments can have adverse effects and it is important to know the likelihood of experiencing such adverse effects when recommending a treatment. The adverse effects for the four most effective AED (valproic acid, lamotrigine, levetiracetam, ethosuximide) and non-AED (ketogenic diet, Atkin's or modified Atkin's diet, gluten free casein free diet and hyperbaric oxygen therapy) treatments for clinical seizures were reviewed. The survey asked the parents to state the specific adverse effects, up to three, associated with each treatment and to rate each of the adverse effects as mild, moderate or severe. The rates of reporting one, two or three adverse effects regardless of the severity and for severe adverse effects only for each treatment are presented in Table 10. In general, AED treatments, except for ethosuximide, were reported to have a higher rate of adverse effects as compared to non-AED treatments, especially with respect to severe adverse effects. Ethosuximide, an AED treatment, and the ketogenic diet, a non-AED treatment, demonstrated slightly higher rates of adverse effects as compared to the non-AED treatment besides the ketogenic diet.

**Table 10:** Rates of adverse effects regardless of the severity and the rate of severe adverse effects for treatments perceived to be most effective.

	Number of Adverse Effects			Number of Severe Adverse Effects		
Treatment	One	Two	Three	One	Two	Three

Valproic Acid	48%	25%	11%	15%	4%	5%

Lamotrigine	34%	19%	12%	16%	3%	1%

Levetiracetam	43%	19%	10%	16%	2%	3%

Ethosuximide	26%	7%	7%	7%	0%	4%

Ketogenic Diet	27%	13%	4%	8%	0%	2%

Atkin's Diet/Modified Atkin's Diet	15%	0%	0%	0%	0%	0%

Gluten-free Casein-free Diet	6%	2%	1%	3%	1%	0%

Hyperbaric Oxygen Therapy	10%	6%	4%	6%	0%	2%

The rates for specific adverse effects are outlined in Table 11-12 for the eight treatments described above. AED treatments (Table 11) demonstrated a higher rate of mood and behavioral changes as compared to non-AED treatments (Table 12). AED treatments and the ketogenic and Atkin's/modified Atkin's diets tended to result in drowsiness, tiredness or fatigue. The ketogenic diet also appeared to result in a high rate of constipation or diarrhea.

**Table 11:** Rates of specific adverse effects for the antiepileptic drug treatments reported to be most effective

Adverse Effects	Valproic Acid	Lamotrigine	Levetiracetam	Ethosuximide
Mood Instability or Anger	10%	10%	21%	4%

Behavioral Change	5%	3%	8%	0%

Drowsiness/tiredness/fatigue	10%	4%	8%	7%

Change in appetite or weight	11%	2%	3%	7%

Increase in seizures	2%	5%	4%	7%

Constipation or diarrhea	3%	2%	1%	0%

Sleep disruption	4%	5%	3%	4%

Inattention or confusion	4%	3%	4%	0%

Rash or allergic reaction	1%	7%	1%	0%

Hyperactivity	4%	3%	3%	4%

Nausea and/or vomiting	2%	3%	1%	4%

Ataxia, tremor or dizziness	6%	3%	1%	0%

Unusual infection	1%	0%	1%	0%

Movement disorder	1%	2%	2%	0%

Anxiety	1%	1%	2%	0%

Headache	1%	3%	0%	0%

Hair loss	3%	1%	0%	0%

Visual disturbances	0%	1%	1%	0%

Speech disturbance	3%	4%	4%	0%

Enuresis	1%	0%	1%	0%

Other	6%	0%	2%	4%

**Table 12:** Rates of specific adverse effects for the non-antiepileptic drug treatments reported to be most effective

Adverse Effects	Ketogenic Diet	Atkin's or modified Atkin's Diet	Gluten-free Casein-free Diet	Hyperbaric Oxygen Therapy
Mood Instability or Anger	0%	0%	2%	4%

Behavioral Change	2%	0%	1%	2%

Drowsiness/tiredness/fatigue	8%	0%	1%	0%

Change in appetite or weight	2%	10%	2%	0%

Increase in seizures	4%	0%	0%	0%

Constipation or diarrhea	12%	5%	3%	0%

Sleep disruption	2%	0%	0%	4%

Inattention or confusion	0%	0%	1%	2%

Rash or allergic reaction	0%	0%	0%	0%

Hyperactivity	2%	0%	0%	0%

Nausea and/or vomiting	2%	0%	0%	0%

Ataxia, tremor or dizziness	0%	0%	0%	0%

Unusual infection	0%	0%	0%	4%

Movement disorder	2%	0%	0%	0%

Anxiety	0%	0%	0%	0%

Headache	0%	0%	0%	0%

Hair loss	0%	0%	0%	0%

Visual disturbances	0%	0%	0%	0%

Speech disturbance	0%	0%	0%	0%

Enuresis	0%	0%	0%	0%

Other	10%	0%	0%	2%

## Discussion

This study examined the clinical characteristics of children with ASD and clinical and/or subclinical seizures, their management, and parental perception of effectiveness of traditional treatments for seizures and non-traditional treatments using an on-line survey. A survey that gathered information about children with ASD without seizures was also used to gather control data. The surveys were advertized on ASD-related websites. Both traditional treatments for seizures and non-traditional ASD treatments were considered and the perceived effectiveness of these treatments on seizures, sleep, communication, behavior, attention and mood was queried. The perceived effectiveness of treatments was analyzed for individuals with clinical and subclinical seizures separately. One of the most important findings of this study was that certain AED and non-AED treatments were perceived as improving seizures while also improving, or at least not worsening, other clinical factors that are important in children with ASD. We will address specific aspects of this study's findings in separate sections below.

### Characteristics of Children with ASD and Seizures

The proportion of male children in both the seizures and control survey was exactly the same, 77%, and consistent with other reports of the portion of males in the general ASD population [19]. Children with ASD and both clinical and subclinical seizures were more likely to have a diagnosis of Autism Disorder, and less likely to have a diagnosis of Asperger syndrome, than the control children. Children with ASD and clinical seizures, but not subclinical seizures, were more likely to have mental retardation than control children but other medical conditions were not different between the seizure and control groups. This suggests that children with ASD and clinical seizures have more severe cognitive difficulties as compared to children with ASD without seizure, consistent with previous studies [20-22]. Children with ASD and both clinical and subclinical seizures are more likely to have regression as compared to control children, consistent with previous studies [23]. It is important to point out that despite the higher rate of regression in children with ASD and either clinical or subclinical seizures, the majority of such children in these groups did not have regression. Thus, it is important to approach children with both regressive and non-regressive ASD with a similar index of suspicion when considering a workup for seizures.

### Management and Diagnosis of Children with ASD and Seizures

Overall, for children with ASD in general, pediatricians managed medical and developmental issues. A child neurologist managed the medical and developmental issues of the majority of children with ASD and either clinical and/or subclinical seizures. The medical and developmental aspects of the child were managed, in part, by a doctor affiliated with Defeat Autism Now! in approximately one-third of children overall. It is clear from this data that multiple practitioners, in general, manage children with ASD, presumably due to the complexity of this disorder.

The great majority of children (i.e., > 88%) with clinical and subclinical seizures were diagnosed and managed, at least in part, by a child or adult neurologist. This is an encouraging finding, but it is important for all children with any type of seizure activity to be managed by an expert in seizures such as a neurologist or epileptologist. Over half of the control children with ASD were never evaluated for seizures. Our recent study on subclinical discharges demonstrated that many of the children with such discharges have no specific symptoms except for delays in language development and features of ASD and/or attention deficit [24]. Thus, it is important to keep a high index of suspicion for seizures, especially subclinical seizures, in children with ASD since the symptoms of subclinical seizures can be subtle [24]. In addition, partial complex seizures, especially that originate in the temporal or frontal lobes can present as complex behaviors and automatisms that may be difficult to differentiate ASD behaviors [25-30]. This may be true especially in non-verbal and/or non-communicative children who cannot express symptoms and in whom paradoxical symptoms, such as speech arrest, cannot be easily observed.

The majority of children with ASD and seizures underwent a routine EEG as a diagnostic test for their clinical and/or subclinical seizure disorder while significantly fewer children with ASD and clinical seizures had an overnight and ambulatory EEG as compared to children with subclinical seizures. Of course, many of the subclinical seizure disorders require an overnight EEG to diagnose, so this is not unexpected. Of the children with clinical and subclinical seizures, 17% and 10% of them did not have any diagnostic test for diagnosis, respectively. This is concerning, especially for subclinical seizures, since ASD behaviors, especially staring, can be mistaken for subtle seizure activity.

### Treatments for Clinical Seizures

One of the most important findings of this study was that AED treatments, as compared to non-AED treatments, were perceived by parents as improving seizures but, as a group, they were perceived as worsening other clinical factor (i.e., sleep, communication, behavior, attention and mood) for children with ASD and clinical seizures. However, four AED treatments (valproic acid, lamotrigine, levetiracetam and ethosuximide) were perceived by parents to improve seizures and not worsen other clinical factors. Clearly AEDs are important for treating clinical seizures in children with ASD but do not appear to be rated as helpful for treating other comorbid clinical factors associated with ASD such as communication, behavior and mood. It is somewhat surprising that AEDs such as valproic acid and lamotrigine were not perceived to have a more positive effect on mood as both of these medications are considered mood stabilizers [6].

Interestingly, most AED and non-AED treatments examined were perceived to improve seizures or other clinical factors, but not both. The exception were four non-AED treatments (ketogenic diet, Atkin's or modified Atkin's diet, gluten free casein free diet and hyperbaric oxygen therapy), which were perceived to improve both seizures and other clinical factors. ASD has been suggested to be associated with cortical hyperexcitability [5]. AEDs exert their effect by reducing cortical excitability [31]. The fact that some of the key symptoms associated with ASD (i.e., communication and behavior) are perceived to be improved by reducing cortical hyperexcitability suggests that the cortical hyperexcitability associated with seizures is not a major part of the neuropathology leading to the cognitive features associated with ASD, at least in the subgroup of children with clinical seizures. Interestingly, although the cortical hyperexcitability associated with ASD has been attributed to an inhibitory deficit [5], many of the AEDs thought to target inhibitory gamma-aminobutyric acid mechanisms were not the ones perceived to be most effective in this population.

Many parents reported using non-AED treatments for their children with ASD and seizures. Interestingly, several non-AED treatments were perceived as efficacious in treating both seizures and other clinical factors. One treatment, the ketogenic diet, was perceived as very effective for improving seizures as compared to other AED and non-AED treatments and was perceived as having favorable effects on sleep, communication, behavior, attention and mood. The ketogenic diet is well known to be effective in drug resistant epilepsy [32] and has been reported to be effective in the treatment of ASD [33]. The effectiveness of the ketogenic diet for the treatment of both ASD and seizures could be through several mechanisms. The ketogenic diet has been shown to be effective for improving mitochondrial function [34] and oxidative stress [35], two biological mechanisms believed to underlie the neuropathology associated with both ASD [11,36,37] and seizures [13]. The rate of adverse effects, especially for multiple adverse effects, was lower for ketogenic diet as compared to the AED treatments and the specific adverse effect profile was different with lower rates of adverse behavior or mood as compared to AED treatments. The Atkin's or modified Atkin's diet which, like the ketogenic diet, promotes ketosis and limits carbohydrate metabolism, was also perceived as effective at improving seizures with a favorable adverse effect profile and has been reported to have similar effectiveness at treating epilepsy as the ketogenic diet [32]. Of course the ketogenic diet, as well as any dietary treatment, can result in adverse effects, such as severe acidosis, which might dangerous in children with ASD, particularly those with metabolic conditions such as mitochondrial disorders. Thus, the ketogenic diet as well as other therapies should be managed by a practitioner with considerable experience with such therapies. Clearly these treatments require further study in individuals with ASD and seizures.

### Treatments for Subclinical Seizures

For the subclinical seizure group, AED treatments were not perceived to significantly improve cognitive and behavioral factors related to ASD. This was somewhat of a surprise as some of the AEDs reported to be used in this group have been associated with improvement in language, attention and behavior in some children with subclinical seizures, particularly those with Landau-Kleffner syndrome [38] and subclinical epileptiform discharges [24]. The ketogenic diet and steroids were in the non-AED treatment cluster that was rated as showing improvement in communication and behavior, thereby verifying previous studies [38,39]. Overall, the ketogenic diet and steroids were perceived as having a similar benefit as compared to several other non-AED treatments (see Table 8 and Figure 3), but the perceived improvement attributed to treatments within this cluster, on average, was not particularly high. This is consistent with the very variable effectiveness of treatments for subclinical epileptiform discharges [38]. Most likely, a more detailed approach to analyzing this group of patients by, perhaps, examining individual patient-to-patient variability, will provide more information regarding which patients respond to treatment. A larger number of respondents would also assist in providing more accurate information.

### Limitations

This study was designed to obtain general parental impressions regarding the effectiveness of seizure specific treatments and general alternative ASD treatments on seizures and other clinical factors. In this study we consider each treatment in isolation as if it was given in a controlled manner and any resulting effects could be directly related to the treatment. However, many children with ASD are prescribed multiple treatments and many families initiate and/or discontinue treatments without advice from the practitioner that is managing their child. Despite this limitation, some clear perceived differences between treatments were evident in this survey.

It should be mentioned that the relation to the individual being reported was not queried, so it is possible that other caretakers of a child with ASD could have completed the survey instead of the parents. The information obtained was only the perception of the parents and was not documented by trained professionals. This may have limited the validity of the information in several ways. First, instruments used to diagnose ASD vary from clinic-to-clinic and it is possible that the ASD diagnosis was not made using any standardized tool since we did not verify the diagnosis. However, the overwhelming majority of parents reported that either a child or adult neurologist managed their ASD child if they had clinical seizures, subclinical epileptiform discharges or seizure-like activity and the majority of the parents of children with ASD who completed the control survey reported that the a pediatrician or psychiatrist managed their child's health and development. Thus, the parents who responded to this survey had adequate professional advice on which to base their report.

This study is subject several other limitations including potential bias of the responders. Most concerning is that individuals who tend to use non-traditional therapies may be critical of traditional drug treatments due to adverse effects [10]. So, relative rankings of one AED vs. another may be reasonable, but the effectiveness of AEDs may be somewhat under-reported relative to non-AED treatments. In addition, since medical information was obtained from parents, not medical records, this information could contain inaccuracies. Furthermore, the diagnosis of the subclinical seizures types can be very variable from practitioner-to-practitioner and some individuals may be told that an EEG is normal if it contains paroxysmal discharges that are considered to be not clinically significant by the practitioner. Despite these limitations, the information gained from this survey provides important insight into which treatments should be further evaluated for individuals with ASD and clinical seizures.

Only 6% of children, overall, were reported to have genetic conditions, which is at the lower end of some estimates of the number of children with ASD who have genetic conditions [40]. This suggests that we may have under sampled this subgroup of children with ASD.

## Conclusions

Parents of children with ASD report that AEDs improve seizure control but worsen other clinical factors in individuals with ASD and clinical seizures. Particular AEDs, including valproic acid, lamotrigine, levetiracetam, are reported to provide the best seizure control and worsen sleep, communication, behavior, attention and mood the least out of all of the AEDs in children with ASD and clinical seizures. In individuals with ASD and clinical seizures, non-AED treatments, in general, were reported to improve sleep, communication, behavior, attention and mood but improve seizures significantly less than AED treatments. Particular non-AED treatments, such as the ketogenic diet, were perceived to improve both seizures and other cognitive and behavioral factors in individuals with ASD and clinical seizures. Although this survey-based method only provides information of the parents' perceptions of effectiveness, this information is important for selecting seizure treatments in individuals with ASD that should undergo further evaluation.

## Competing interests

The authors declare that they have no competing interests.

## Authors' contributions

All authors read and approved the final manuscript. REF designed and created the survey, directed and managed data analysis, performed the statistical analysis and wrote the paper. SS performed the data analysis under the direction of REF. JBA helped design and create the survey and edit the paper.

## Authors' Information

Dr. Richard E. Frye received his medical degree from Georgetown University. He completed his pediatric residency training at Jackson Memorial Hospital/University of Miami and child neurology residency training at Children's Hospital Boston/Harvard University. Following residency Dr. Frye completed concurrent fellowships, one in Behavioral Neurology and Learning Disabilities at Children's Hospital Boston/Harvard University and another in Psychology at Boston University. Dr. Frye completed a Ph.D. in Physiology and Biophysics at Georgetown University and a M.S. in Biomedical Science and Biostatistics at Drexel University. Dr. Frye is board certified in Pediatrics and in Neurology with special competency in Child Neurology. Dr. Frye has been funded by the National Institutes of Health to study brain function in individuals with neurodevelopmental disorders. Dr. Frye is the medical-director of the University of Texas medically-based autism clinic. The purpose of this unique clinic is to diagnose and treat medical disorders associated with autism in order to optimize remediation and recovery.

Swapna Sreenivasula is currently a graduate student in the School of Public Health at University of Texas. She completed a Bachelor of Medicine and Bachelor of Surgery (M.B.B.S.) at Rangaraya Medical College, Kakinada Andhra Pradesh, India.

James B. Adams, Ph.D., is currently a President's Professor and Program Chair of Materials Engineering at Arizona State University, and he directs the Autism/Asperger's Research Program at Arizona State University. He created and teaches a course on Heavy Metal Toxicity, focused on lead and mercury toxicity. He co-leads the Science Advisory Committee of the Autism Research Institute. He is the father of a young woman with autism, and that is what led him to eventually shift much of his research emphasis to autism, focusing on biological causes and treatments.

## List of abbreviations

ASD: Autism Spectrum Disorder; ARI: Autism Research Institute; EEG: Electroencephalograph; PDD-NOS: Pervasive Developmental Disorder-Not Otherwise Specified;

## Pre-publication history

The pre-publication history for this paper can be accessed here:

http://www.biomedcentral.com/1471-2431/11/37/prepub

## Supplementary Material

Additional file 1 **Appendices**. List of participants of the Elias Tembenis Seizures Think Tank and Invitation Letter for Survey Click here for file

Additional file 2 **Tables S1-S6**. Tables containing statistical values from the analyses of variance. Click here for file
