# Supplementary material for: Traditional and non-traditional treatments for autism spectrum disorder with seizures: an on-line survey
Source: BMC Pediatr. 2011 May 18;11:37. doi: 10.1186/1471-2431-11-37 (PMC3123184; doi:10.1186/1471-2431-11-37)
Supplement: Additional file 1 — Appendices. List of participants of the Elias Tembenis Seizures Think Tank and Invitation Letter for Survey [file 1471-2431-11-37-S1.DOC]

Appendix A: Participants of the Elias Tembenis Seizures Think Tank

| **Participant** | **Specialty** |
| --- | --- |
| **Richard Frye, MD, PhD** | **Child and Behavioral Neurology** |
| **James Adams, PhD** | Mechanical, Aerospace, Chemical, and Materials Engineering |
| Derrick MacFabe, MD | **Neurology, Neurophysiology** |
| Manuel Casanova, MD | **Neuropathology** |
| Paul Hardy, MD | **Child Neurology** |
| Jeffrey Lewine, PhD | **Neuropsychology, Neurophysiology** |
| Maya Shetreat-Klein, MD | **Child Neurology** |
| Tapan Audhya, PhD | **Vitamin Supplementation** |
| **Gregory Brown, MD** | **Alternative Medicine, Mineral Supplementation** |
| Vicki Martin, RN | **Alternative Medicine, Mineral Supplementation** |
| **Rob Coben, PhD** | **Neuropsychology, Neurophysiology** |
| **Stephen Edelson, PhD** | **Alternative Medicine** |
| **Seyyed Hossein Fatemi, MD, PhD** | Psychiatry, Cell Biology and Neuroanatomy |
| **Cindy L. Griffin, DSH-P, DIHom** | Homeopathy |
| Lindyl Lanham, DSH-P, HD | Homeopathy |
| Jon Poling, MD, PhD | **Neurology, Neurophysiology** |
| Dan Rossignol, MD | **Alternative Medicine** |
| Harry Schneider, MD | **Neuroimaging** |
| **Allan Sosin, MD** | **Alternative Medicine** |
| Theoharis Theoharides, PhD | Pharmacology, Internal medicine, Biochemistry, Immunology |
| Aristo Vojdani, PhD | Immunology |

**Appendix B.** Invitation Letter for Survey

Dear  Autism family,

There is little understanding of the treatments that help control seizures and seizure-like activity in children with autism. To help understand which treatments are effective for controlling seizures, seizure-like activity and subclinical epileptiform discharges in autism Dr. Richard Frye has developed a relatively easy to complete on-line seizure survey for parents with children with autism spectrum disorder with and without seizures to complete. It is important that we obtain information about individuals without seizures, so that we can determine whether treatments affect individuals with and without seizures differently.

If your son/daughter suffers from autism spectrum disorder, regardless of whether you have clinical seizures, subclinical epileptiform discharges or seizure-like activity, we would like to ask you to fill out a survey created by Dr. Richard Frye. We think that this very detailed survey will provide a much richer understanding of the efficacy and possible side-effects of current treatments for seizures.

Please help us by filling out the survey and by forwarding information about this survey to other autism groups.

For individuals with seizures, subclinical epileptiform discharges or seizure-like activity, please use the following link:

<http://www.surveymethods.com/EndUser.aspx?A286EAF0A7E6FEF0A0>

For individuals without seizures, subclinical epileptiform discharges or seizure-like activity, please use the following link:

<http://www.surveymethods.com/EndUser.aspx?D9FD918BDC9D858AD2>

Thank you very much for helping us find answers to seizures in autism.

Sincerely,

Richard E. Frye, M.D., Ph.D., F.A.A.P.

Assistant Professor of Pediatrics and Neurology

University of Texas-Houston
