# Supplementary material for: Traditional and non-traditional treatments for autism spectrum disorder with seizures: an on-line survey
Source: BMC Pediatr. 2011 May 18;11:37. doi: 10.1186/1471-2431-11-37 (PMC3123184; doi:10.1186/1471-2431-11-37)
Supplement: Additional file 2 — Tables S1-S6. Tables containing statistical values from the analyses of variance. [file 1471-2431-11-37-S2.DOC]

Table S1. F-values for the analysis of variance for each clinical characteristic queried by the survey for the Tier 1 analysis. Note that the effect of seizure type and interaction between seizure type and group were not significant in the analysis of variance and, thus, are not shown. There were 12 omnibus analyses, one for each characteristic. The Bonferroni correction results in an alpha of 0.05/12 = 0.004, so the alpha was set at p<=0.001 to be conservative.

| Characteristic | F-values for Tier 1 Cluster Effect |
| --- | --- |
| Seizures | 25.49† |
| Sleep | 173.15† |
| Communication | 584.12† |
| Receptive | 550.02† |
| Expressive | 521.59† |
| Verbal | 519.27† |
| Nonverbal | 533.92† |
| Behavior | 432.95† |
| Stereotype Movements | 389.17† |
| Rigidity | 428.43† |
| Hyperactivity | 220.06† |
| Attention | 626.04† |
| Mood | 420.74† |

†p<0.0001

Table S2. Statistical values for the analysis of variance and planned contrasts for each clinical characteristic queried by the survey for the Tier 2 analysis of the AED treatment cluster. Note that the effect of seizure type and interaction between seizure type and group were not significant in the analysis of variance and, thus, are not shown. Since there were 12 omnibus analyses, one for each characteristic. The Bonferroni correction results in an alpha of 0.05/12 = 0.004, so the alpha was set at p<=0.001 to be conservative. For the planned contrasts, there are 3 comparisons for each analysis. The Bonferroni correction results in an alpha of 0.05/3 = 0.017, so the alpha was set at p<=0.01 to be conservative.

| Characteristic | F-values for AED Subcluster Effect | T-value for contrasts | | |
| --- | --- | --- | --- | --- |
|  |  | Subcluster 1 v  Subcluster 2 | Subcluster 1 v  Subcluster 3 | Subcluster 2 v  Subcluster 3 |
| Seizures | 11.92† | 4.84† | 0.72 | 1.56 |
| Sleep | 1.04 |  |  |  |
| Communication | 25.73† | 4.39† | 6.65† | 4.70† |
| Receptive | 19.89† | 3.48‡ | 6.01† | 4.47† |
| Expressive | 31.97† | 4.93† | 7.40† | 5.20† |
| Verbal | 17.63† | 3.59‡ | 5.53† | 3.93† |
| Nonverbal | 24.31† | 4.55† | 6.32† | 4.28† |
| Behavior | 8.17† | 0.52 | 4.00† | 3.83† |
| Stereotype | 10.03† | 1.16 | 4.48† | 4.01† |
| Rigidity | 4.92 | 1.57 | 3.04€ | 2.35 |
| Hyperactivity | 6.90‡ | 1.30‡ | 3.00€ | 3.67‡ |
| Attention | 19.92† | 2.80€ | 6.21† | 5.00† |
| Mood | 4.75 | 0.83 | 3.08€ | 2.74€ |

€p<=0.01; ‡p<=0.001; †p<=0.0001

Table S3. Statistical values for the analysis of variance and planned contrasts for each clinical characteristic queried by the survey for the four AED treatments in AED subcluster 1 derived from the Tier 2 cluster analysis. Note that the effect of seizure type and interaction between seizure type and treatment were not significant in the analysis of variance and, thus, are not shown. There were 12 omnibus analyses, one for each clinical characteristic. The Bonferroni correction resulted in an alpha of 0.05/12 = 0.004, so the alpha was set to p<=0.001 to be conservative. For the planned contrasts, there are 6 comparisons for each analysis. The Bonferroni correction resulted in an alpha of 0.05/6 = 0.008, so the alpha was set to p<=0.001 to be conservative (VPA = valproic acid; LTG = lamotrigine; LVT = levetiracetam; ETH = ethosuximide).

| Clinical  Characteristic | F-Value for Treatment  Effect | T-value for planned contrasts | | | | | |
| --- | --- | --- | --- | --- | --- | --- | --- |
|  |  | VPA v  LTG | VPA v  LVT | VPA v  ETH | LTG v  LVT | LTG v  ETH | LVT v  ETH |
| Seizures | 1.83 |  |  |  |  |  |  |
| Sleep | 0.60 |  |  |  |  |  |  |
| Communication | 8.80† | 5.08† | 2.23 | 1.78 | 2.57 | 0.65 | 0.67 |
| Receptive | 11.01† | 5.53† | 2.88 | 2.63 | 2.36 | 0.04 | 1.18 |
| Expressive | 8.95† | 5.18† | 2.33 | 1.07 | 2.57 | 1.42 | 0.08 |
| Verbal | 8.52† | 4.99† | 2.35 | 1.81 | 2.36 | 0.58 | 0.65 |
| Nonverbal | 4.85 |  |  |  |  |  |  |
| Behavior | 4.73 |  |  |  |  |  |  |
| Stereotype | 1.55 |  |  |  |  |  |  |
| Rigidity | 6.08‡ | 3.56‡ | 0.67 | 0.62 | 3.87† | 1.07 | 0.93 |
| Hyperactivity | 4.04 |  |  |  |  |  |  |
| Attention | 6.26‡ | 4.20† | 1.80 | 1.91 | 2.15 | 0.10 | 1.01 |
| Mood | 9.39† | 2.47 | 3.25‡ | 0.32 | 5.28† | 0.85 | 1.86 |

‡p<=0.001; †p<=0.0001

Table S4. Statistical values for the analysis of variance and planned contrasts for each clinical characteristic queried by the survey for the Tier 2 analysis of the non-AED treatment cluster. Note that the effect of seizure type and interaction between seizure type and subcluster were not significant in the analysis of variance and, thus, are not shown. There were 12 omnibus analyses, one for each characteristic. The Bonferroni correction resulted in an alpha of 0.05/12 = 0.004, so the alpha was set at p<=0.001 to be conservative. For the planned contrasts, there are 3 comparisons for each analysis. The Bonferroni correction resulted in an alpha of 0.05/3 = 0.017, so the alpha was set at p<=0.01 to be conservative.

| Characteristic | F-Value for Non-AED  Subcluster Effect | T-value for contrasts | | |
| --- | --- | --- | --- | --- |
|  |  | Subcluster 1 v  Subcluster 2 | Subcluster 1 v  Subcluster 3 | Subcluster 2 v  Subcluster 3 |
| Seizures | 34.28† | 7.13† | 1.33 | 5.37† |
| Sleep | 40.38† | 8.84† | 4.69† | 0.04 |
| Communication | 49.80† | 9.33† | 6.68† | 1.87 |
| Receptive | 38.48† | 8.29† | 5.66† | 1.38 |
| Expressive | 48.33† | 9.13† | 6.70† | 2.00 |
| Verbal | 44.18† | 8.72† | 6.39† | 1.94 |
| Nonverbal | 49.27† | 9.30† | 6.58† | 1.78 |
| Behavior | 64.77† | 10.39† | 8.08† | 2.76€ |
| Stereotype | 58.60† | 10.20† | 7.06† | 1.79 |
| Rigidity | 46.15† | 8.45† | 7.31† | 3.03€ |
| Hyperactivity | 49.83† | 9.11† | 7.11† | 2.45€ |
| Attention | 51.57† | 9.70† | 6.31† | 1.27 |
| Mood | 62.61† | 8.99† | 9.46† | 5.01† |

€p<0.01; ‡p<0.001; †p<0.0001

Table S5. Statistical values for the analysis of variance and planned contrasts for each clinical characteristic queried by the survey for the four non-AED treatments in non-AED subcluster 1 derived from the Tier 2 cluster analysis. Note that the effect of seizure type and interaction between seizure type and treatment were not significant in the analysis of variance and, thus, are not shown. There were 12 omnibus analyses, one for each clinical characteristic. Since the Bonferroni correction resulted in an alpha of 0.05/12 = 0.004, the alpha was set to p<=0.001 to be conservative. For the planned contrasts, there are 6 comparisons for each analysis. The Bonferroni correction resulted in an alpha of 0.05/6 = 0.008, so the alpha was set to p<=0.001 to be conservative (KD = ketogenic diet, AD = Atkin’s or modified Atkin’s diet, GFCF = gluten-free casein-free diet, HBOT = hyperbaric oxygen therapy).

| Characteristic | F-Value for Treatment Effect | T-value for planned contrasts | | | | | |
| --- | --- | --- | --- | --- | --- | --- | --- |
|  |  | KD v  AD | KD v  GFCF | KD v  HBOT | AD v  GFCF | AD v  HBOT | GFCF v  HBOT |
| Seizures | 16.06† | 3.11 | 5.25† | 6.78† | 0.27 | 2.11 | 3.22‡ |
| Sleep | 5.12 |  |  |  |  |  |  |
| Communication | 1.76 |  |  |  |  |  |  |
| Receptive | 1.84 |  |  |  |  |  |  |
| Expressive | 2.61 |  |  |  |  |  |  |
| Verbal | 2.32 |  |  |  |  |  |  |
| Nonverbal | 1.25 |  |  |  |  |  |  |
| Behavior | 11.76† | 1.72 | 1.02 | 3.56‡ | 2.45 | 1.04 | 5.64† |
| Stereotype | 6.05‡ | 1.44 | 0.73 | 2.49 | 1.96 | 0.52 | 3.97† |
| Rigidity | 7.33† | 1.04 | 1.24 | 2.50 | 1.89 | 0.89 | 4.50† |
| Hyperactivity | 14.27† | 1.98 | 0.72 | 4.27† | 2.52 | 1.32 | 6.21† |
| Attention | 1.88 |  |  |  |  |  |  |
| Mood | 4.87 |  |  |  |  |  |  |

‡p<=0.001; †p<=0.0001

Table S6. F-values for the analysis of variance conducted on each clinical characteristic queried by the survey for the subclnical seizure group. Note that the effect of seizure type and interaction between seizure type and cluster were not significant in the analysis of variance and, thus, are not shown. There were 11 omnibus analyses, one for each characteristic. The Bonferroni correction resulted in an alpha of 0.05/11 = 0.005, so the alpha was set to p<=0.001 to be conservative.

| Characteristic | F-values for Cluster Effect |
| --- | --- |
| Sleep | 10.52‡ |
| Communication | 32.09† |
| Expressive | 32.41† |
| Receptive | 30.86† |
| Verbal | 29.38† |
| Nonverbal | 22.10† |
| Behavior | 13.57‡ |
| Stereotype | 17.13† |
| Rigidity | 24.29† |
| Hyperactivity | 22.45† |
| Attention | 34.80† |
| Mood | 24.31† |

‡p<=0.001; †p<=0.0001
